# Egg Consumption and Mortality: A Prospective Cohort Study of Australian Community-Dwelling Older Adults

**DOI:** 10.3390/nu17020323

**Published:** 2025-01-17

**Authors:** Holly Wild, Danijela Gasevic, Robyn L. Woods, Joanne Ryan, Rory Wolfe, Yuquan Chen, Thara Govindaraju, John J. McNeil, Tracy McCaffrey, Lawrence J. Beilin, Dragan Ilic, Alice J. Owen

**Affiliations:** 1 School of Public Health and Preventive Medicine, Monash University, Melbourne, VIC 3004, Australia; holly.wild@monash.edu (H.W.);; 2 Centre for Global Health, Usher Institute, The University of Edinburgh, Edinburgh EH1 2QZ, UK; 3 Department of Nutrition, Dietetics and Food, Monash University, Notting Hill, VIC 3168, Australia; 4 Medical School, University of Western Australia, Perth, WA 6009, Australia

**Keywords:** eggs, egg consumption, nutrition, older adults, healthy ageing

## Abstract

Background/Objectives: Egg consumption in adults has been linked with a modestly increased risk of all-cause and CVD mortality. However, evidence on adults aged 65 y+ is limited. The objective of this study was to investigate the association between egg intake and mortality in community-dwelling older adults. Methods: In this prospective cohort study, 8756 adults aged 70+ years, participants in the ASPirin in Reducing Events in the Elderly (ASPREE) Longitudinal Study of Older Persons, self-reported the frequency of their total egg intake: never/infrequently (rarely/never, 1–2 times/month), weekly (1–6 times/week), and daily (daily/several times per day). All-cause and cause-specific (cardiovascular disease [CVD] and cancer) mortality was established from at least two sources: medical records, death notices, next of kin, or death registry linkage. The association between egg intake and mortality was assessed using Cox proportional hazards regression analysis, adjusted for socio-demographic, health-related, and clinical factors and overall dietary quality. Results: Over the median 5.9-year follow-up period, a total of 1034 all-cause deaths (11.8%) were documented. A 29% lower risk of CVD mortality (HR (95% CI): 0.71 [0.54–0.92]) and a 17% (HR (95% CI): 0.83 [0.71–0.96]) lower risk of all-cause mortality were observed among those who consumed eggs weekly, compared to those who consumed eggs never/infrequently; no statistically significant association was observed for weekly consumption and cancer mortality. In contrast, compared to those that never or infrequently consumed eggs, daily consumption had slightly higher odds of mortality, though these results did not reach statistical significance. Conclusions: The consumption of eggs 1–6 times per week was associated with a lower risk of all-cause mortality and CVD mortality in community-dwelling adults aged 70 years and over. These findings may be important to inform the development of evidence-based guidelines for egg consumption.

## 1. Introduction

Globally, the proportion of adults aged 65 years and over is increasing, with the number of people aged 80 years and over expected to triple between 2020 and 2025 [1]. Ageing is associated with a gradual loss of lean muscle mass, which can impair function and strength, impacting older adults’ ability to perform activities of daily living [2].

Protein is an essential macronutrient, providing amino acids and nitrogen, which are required for tissue growth and maintenance, as well as being a source of energy. In older adults, low protein intake is associated with a higher risk of sarcopenia [2], and a high intake is associated with greater muscle mass, improved physical performance [3], and a lower risk all-cause mortality [4,5].

Economic challenges, coupled with age-related decline in oral health, can contribute to a reduced consumption of protein-rich food sources in later life [6,7]. Observational research in older cohorts has indicated that the consumption of eggs may overcome some of these reported barriers, as eggs can be an economical, accessible, efficient source of essential nutrients [8,9]. Eggs are also a preferred source of nutrients for older adults experiencing age-related physical and sensory decline [8,10].

Eggs are a rich source of protein and a good source of essential nutrients, such as B vitamins, folate, unsaturated fatty acids, fat-soluble vitamins (E, D, A, and K), choline, and numerous minerals and trace elements [11]. While eggs are commonly considered nutrient-dense, they are also a source of dietary cholesterol [11,12]. This, alongside significant methodological inconsistencies in the epidemiological research on the association between egg consumption and health, such as a lack of a controlling for dietary patterns and quality [13,14], has led to decades-long debate over the risks and benefits of egg consumption [15,16].

The relationship between egg consumption and health has been widely studied, but the findings are often conflicting, making it challenging to establish evidence-based guidelines [11], often leading to inconsistencies between guidelines internationally [17]. Two recent systematic reviews [18,19] of observational studies examining egg consumption and mortality in adults of all ages reported mixed results. Mousavi et al. [19] observed a dose-dependent positive association between egg consumption and all-cause and cancer mortality. Ma et al. [18] indicated a dose-dependent positive association between egg consumption and all-cause mortality for women and CVD mortality risk in specific subgroups, particularly in older cohorts and when adjusting for hyperlipidemia.

Research on the association between egg consumption and mortality in older adults is lacking, with the majority of studies focusing on CVD mortality [18,19]. The findings remain varied in this research and suggest that, while moderate egg consumption may not significantly affect cardiovascular risk in the general population [20,21], a higher intake could pose risks for specific groups, such as postmenopausal women [20]. As eggs have been identified as a key source of accessible protein and nutrition in older adults [8,9], it is important to further explore the benefits and risks associated with consumption.

To address these gaps in the existing research, this study explores the association between egg consumption and both all-cause and cause-specific mortality in older adults (those aged ≥70 y).

## 2. Materials and Methods

### 2.1. Study Population

This secondary analysis utilized data derived from the ASPREE (ASPirin in Reducing Events in the Elderly) trial and one of its prominent sub-studies, the ASPREE Longitudinal Study of Older Persons (ALSOP) study. ASPREE was a double-blind, placebo-controlled, randomized clinical trial that aimed to investigate the impact of daily 100 mg enteric-coated aspirin on the extension of a healthy life in a population of 19,114 older adults residing in Australia (n = 16,703) and the United States (n = 2411) [22,23]. Eligible participants were aged 70 years and older, with the exception of US minority participants who were aged 65 years and older. At enrolment into ASPREE, all participants were free from diagnosed dementia, CVD events, and independence-limiting physical disability and were living independently in the community [22]. The specific inclusion and exclusion criteria for ASPREE have been previously documented [22,24].

ALSOP was a sub-study running alongside the ASPREE trial for Australian participants; thus, only Australian participants were included in this analysis [25]. ALSOP’s primary objective was to investigate various social, behavioral, and environmental factors associated with healthy ageing, and over 85% of Australian ASPREE participants contributed to this sub-study [25]. Following the completion of the active intervention phase, ASPREE participants were further followed up as part of a longitudinal cohort extension study (ASPREE-XT) [26].

ALSOP received ethical approval from the Monash University Human Research Ethics Committee, under project numbers CF11/1100 (14 June 2011) and CF11/1935 (7 October 2011). Meanwhile, ASPREE obtained ethical approval from multiple review boards in both Australia and the USA, with Monash University serving as the primary ethics site in Australia (for further detail refer to https://aspree.org/aus/about-us/ethics-and-privacy/ (accessed on 11 December 2024)). Participants provided signed an informed consent to participate in both the ASPREE and ALSOP studies.

### 2.2. Exposure Variable: Egg Consumption

Egg consumption was evaluated at year 3 of ALSOP, as part of a self-administered 49-item food questionnaire (FFQ) [25], which serves as the origin for the present analysis. Participants were queried regarding their dietary habits with the following question: “*How frequently in the past 12 months did you consume eggs? (boiled, poached or fried)*” Responses were reported using a frequency scale that ranged from *never/rarely; once or twice/month; once or twice/week; often 3–6 times/week; every day or several times a day*. These categories were combined to represent patterns of regular consumption and to preserve power across each of these categories. The final exposure variable included three categories: never or infrequent consumption (“never/rarely” to “once or twice/month”), weekly consumption (“once or twice/week” to “3–6 times/week”), and daily consumption (“every day or several times a day”). The type of egg consumed was not specified in the dietary questionnaire, but the Australian consumption of eggs is mostly chicken eggs [27].

### 2.3. Outcomes

The main outcomes of this study were all-cause mortality (death from any cause), cancer mortality, and CVD mortality. CVD mortality was defined as death from CVD, coronary heart disease, or stroke. Fatal coronary heart disease consisted of death from myocardial infarction, sudden cardiac death, or any other death in which the underlying cause was considered to be coronary heart disease. Fatal stroke was defined as any death in which the underlying cause was a blockage or rupture in the intracranial or extracranial cerebral arterial system. Fatal cardiovascular disease was defined as any death in which the underlying cause was coronary heart disease or stroke [23,24,28].

Information about deaths was collected from two main sources: medical records and notifications from close contacts. The accuracy of reported deaths was checked with a second, independent source, such as the primary care doctor, death notices, or family members. The study also linked to the Australian death registry, the National Death Index [24,28]. Deaths were adjudicated by a committee of expert clinicians for the coding of date and causes of death. Additionally, staff performed weekly linkages to the Ryerson Index [29], a community-maintained registry that monitors death notices and obituaries.

### 2.4. Covariates

Covariates were selected to align with the current evidence on this topic [19]. As dietary data were collected at Year 3 of ALSOP, this was established as the baseline of our study. With the exception of demographic (excluding age) and socio-economic factors measured at ASPREE baseline, and less likely to change over time, all variables were collected at ASPREE/ALSOP Year 3.

Demographic and socio-economic factors included age, sex (male or female), educational attainment (12 years or less and more than 12 years), and area-level socio-economic status, ascertained via residential postcode linkage to the Index of Relative Socio-economic Advantage and Disadvantage (IRSAD) [30] and analyzed as quintiles.

Smoking status was reported as “never”, “former”, or “current” smoker. Regarding alcohol consumption, participants indicated whether they currently consumed alcohol (yes/no). Additionally, they specified the number of standard drinks consumed, with choices including 1, 2, 3, 4, or more than 4 drinks, and also reported the number of days per week on which alcohol was consumed. An aggregate alcohol variable was generated to denote the participants’ alignment with the Australian National Health and Medical Research Council (NHMRC) guidelines [31], which recommend a maximum consumption of no more than 10 standard drinks per week and no more than 4 drinks on any single occasion to mitigate the risk of alcohol-related harm. Alcohol intake was categorized into not current drinkers, alcohol drinking within recommended guidelines, and alcohol consumption in excess of the recommended guidelines, as previous research from the ASPREE cohort has demonstrated that moderate alcohol consumption is associated with reduced mortality risk in comparison to both abstention and excessive consumption [32].

The participants’ weekly physical activity was self-reported (Rare to Never/Low to Moderate/Vigorous). Self-reported oral health status was reported as excellent/very good, good/fair, and poor.

A weighted overall diet quality score was derived responses to the Year 3 ALSOP FFQ. This information encompassed the frequency of consumption of common foods categorized within key food groups and beverages, with a higher score reflecting a better overall dietary quality [33]. The diet quality score was subsequently categorized into tertiles (1: low; 2: moderate; and 3: high).

Biomedical factors were assessed through clinical measurements and self-reported information. These factors included waist circumference, type 2 diabetes, hypertension, and dyslipidemia. Type 2 diabetes was defined as self-reporting, or the use of prescription medication for diabetes, or having a fasting glucose level ≥ 126 mg/dL (≥7 mmol/L). Hypertension was defined as systolic blood pressure ≥ 140 mmHg and/or diastolic blood pressure ≥ 90 mmHg or the use of blood pressure-lowering medication. Dyslipidemia was defined as the reported use of cholesterol-lowering medication or as a serum cholesterol level of at least 212 mg/dL (≥5.5 mmol/L). Self-reported polypharmacy (yes/no) was defined as using 5 or more prescription medications.

Frailty was measured using a 67-item deficit-accumulation frailty index, with each component previously described [34]. The frailty index was calculated for all participants with data for at least 50 items and was the cumulative score across all deficits divided by the number of deficits included. Previous research has identified this index to be a stronger predictor of age-related decline in this cohort than the Fried Frailty Phenotype [34]. Participants were categorized as non-frail (≤0.10), pre-frail (>0.10 and ≤0.21), or frail (>0.21) [34].

Depressive symptoms were assessed using the Center for Epidemiologic Studies Short Depression Scale (CES-D-10) [35], with scores indicating the absence of depressive symptoms (<2), mild depressive symptoms (3–7), or significant depressive symptoms (≥8).

### 2.5. Statistical Analysis

Participant characteristics are presented as mean and standard deviation, if continuous, and counts and percentages if categorical and stratified by egg consumption level. Correlation analyses were conducted to explore potential multicollinearity in the data. No variables correlated at a coefficient higher than 0.7 (the highest correlation was observed between sex and waist circumference (r = 0.38), suggesting no collinearity of data) [36].

A Cox proportional hazards regression analysis was performed to investigate the relationship between egg intake and all-cause mortality. A crude model was performed, as well as a minimally adjusted model [age and sex] and the fully adjusted model [age, sex, IRSAD, quintile education, physical activity, smoking status, alcohol consumption, waist circumference, hypertension, type 2 diabetes, depression score, frailty status, self-reported oral health status, and diet quality score tertiles]. Interactions were tested for all covariates. HRs and 95% CIs were reported.

Acknowledging previous research that suggested the association between egg consumption and mortality may be influenced by dyslipidemia [19] and dietary quality [21,37,38], we performed subgroup analyses for both of these factors. A Fine–Gray competing risk analysis was conducted as a sensitivity analysis to confirm the influence of competing risks and validate the findings of the Cox analysis.

All statistical analyses were conducted using the STATA software, version 17.0 (Stata Corp., College Station, TX, USA) [39].

## 3. Results

### 3.1. Baseline Characteristics

**Figure 1:**
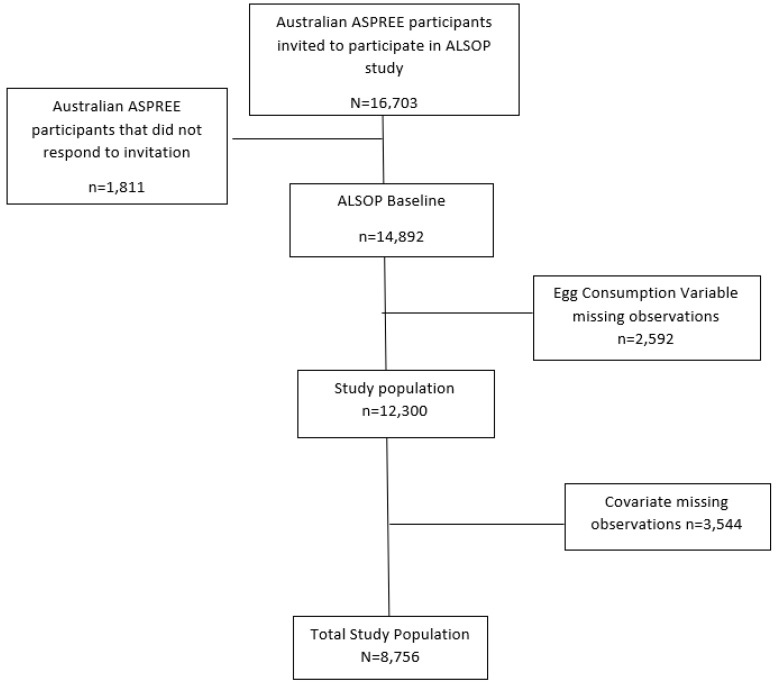
Participant flow chart.

In this prospective analysis, the total study population comprised 8756 individuals (median age of 76.9 ± 5.5 years, 54% women) (Figure 1), of whom 2.6% consumed eggs daily, 73.2% consumed eggs weekly, and 24.2% consumed eggs never/infrequently.

When comparing individuals who consumed eggs never/infrequently to those who consumed eggs weekly or daily, the former group was more likely to be older, have less than 12 years of formal education, to have lower levels of physical activity, to be non-drinkers, and to have a low diet quality score (Table 1).

### 3.2. Egg Consumption and All-Cause and Cause-Specific Mortality

**Figure 2:**
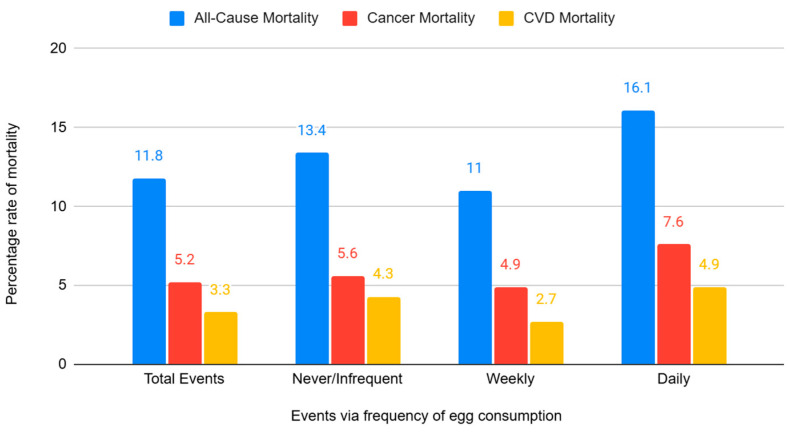
All-cause and cause-specific mortality, total and by frequency of egg consumption.

Over the median 5.9-year follow-up period, a total of 1034 deaths (11.8%) due to all causes were documented, of these 453 (5.2%) were cancer deaths and 229 (3.2%) were CVD deaths (Figure 2; Table S1).

**Figure 3:**
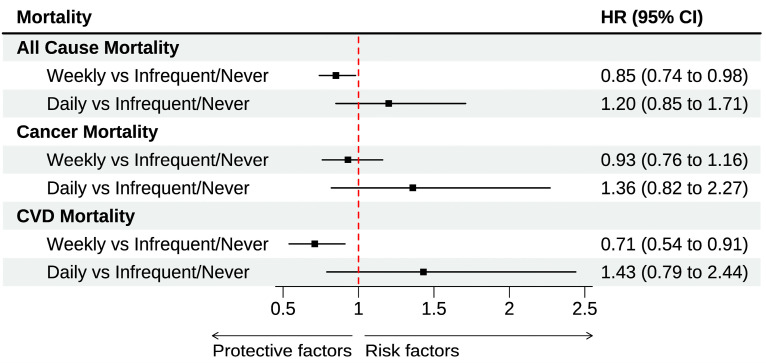
The association between egg consumption and all-cause mortality and cause-specific mortality in community-dwelling older adults: results of the Cox regression.

No interactions were present within the models. Trends observed for weekly and daily consumption were maintained across the crude, minimally, and fully adjusted models for all three outcomes (Table 2). After adjusting for putative confounders compared to those who infrequently/never consume eggs, those who consume eggs weekly (1–6 times per week) had a 15% (HR: 0.85 [0.74–0.98]) lower risk of all-cause mortality and a 29% (HR: 0.71 [0.54–0.91]) lower risk of CVD mortality. No differences in all-cause mortality and CVD mortality risk were observed between people reporting daily egg consumption and those reporting no/infrequent egg consumption. No association was present between egg consumption and cancer mortality (Table 2, Figure 3).

### 3.3. Egg Consumption and All-Cause and Cause-Specific Mortality: Subgroup Analysis

**Figure 4:**
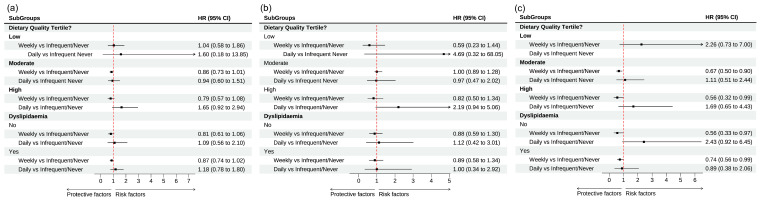
The association between egg consumption and all-cause and cause-specific mortality in community-dwelling older adults: fully adjusted results of the Cox regression stratified by diet quality score and dyslipidemia. HR: Hazard ratio, 95% CI: 95% Confidence intervals. Dietary quality score tertiles (1: low; 2: moderate; 3: high) (**a**): all-cause mortality; (**b**): CVD mortality; (**c**): cancer mortality.

Those who had moderate and high diet quality scores and consumed eggs weekly demonstrated a 33% (HR: 0.67 [0.50–0.90]) and 44% (HR: 0.56 [0.32–0.99]) lower risk of CVD mortality, compared to their counterparts who consumed eggs never/infrequently (Figure 4). No such association was reported for all cause and cancer mortality (Table 3, Tables S2 and S3; Figure 4).

A 27% (HR 0.73 [0.54–0.97]) lower risk of CVD mortality was observed for participants with dyslipidemia and who consumed weekly eggs compared to those who never or infrequently consumed eggs; this risk was further lowered to 43% (HR: 0.57 [0.33–0.97]) for weekly egg consumers without dyslipidemia (Table 3, Tables S2 and S3; Figure 4).

### 3.4. Sensitivity Analysis: Competing Risk Analysis

The results of the competing risk analyses (Fine–Gray) yielded similar results to those of the main models, confirming that the results observed in the specific mortality models are not influenced by competing mortality risk (see Tables S4–S6).

## 4. Discussion

In this prospective cohort study of 8756 adults aged 70 years and over, egg consumption of 1–6 times per week, but not daily egg consumption, was associated with a lower risk of all-cause mortality and CVD mortality. There was no significant association between egg consumption and cancer mortality. As cancer death was not aggregated by cancer type, the lack of association demonstrated in these results may be impacted by variability between cancer types in terms of associations with dietary risk factors.

Our primary results are supported by studies such as Wang and colleagues’ [40] 8-year follow-up of the China Family Panel Study (n = 30,835 adults aged 16–110 years), which reported that moderate egg consumption (3–6 times per week) was associated with a lower risk of all-cause mortality (16%); high consumption (≥7 times/week) further lowered this risk (18%). However, our findings and those of Wang et al. [40] differ from much of the literature in adults, which observes no associations between moderate egg consumption and mortality [41,42,43,44] and a dose-dependent higher risk of mortality with increased egg intake [18,19,43].

Mousavi et al.’s. [19] pooled analysis of 32 studies showed no association between egg consumption and all-cause or CVD mortality but found a positive association with cancer mortality. Their dose-dependent analysis reported a 2% and 4% higher risk of all-cause and cancer mortality, respectively, and a 4% lower risk of stroke mortality, with the additional consumption of 1 egg/week. Darooghegi et al.’s [43] meta-analysis of 55 studies indicated no association between low (0.5 eggs/day) to moderate egg consumption and mortality. However, a daily increase above 0.5 eggs was linked to a 7% higher risk of all-cause mortality and a 13% higher risk of cancer mortality. While our study did not explore dose-dependent associations between egg consumption and mortality, consistent with the findings of Mousavi and colleagues, we did observe that a higher consumption [daily/several times per day] was associated with a higher risk of mortality across primary and subgroup analysis, though these results did not reach statistical significance.

Variations in findings within the evidence base seem to be influenced by demographic, dietary, and health-related factors. In their dose–response meta-analysis of 19 prospective studies, Yang et al. [45] observed a dose-dependent increase in the risk of all-cause (8%), CVD (7%), and cancer (16%) mortality with each additional egg per day. In the subgroup analysis, the all-cause mortality risk was observed to be slightly lower (7%) for those over 60 years of age, suggesting potential age-related differences in risk. In their meta-analysis, Mousavi et al. [19] found an inverse association between egg consumption and mortality in studies from Asia compared to US studies and suggested that these associations may be confounded by the diet and lifestyle patterns associated with egg consumers in western countries, who are more likely to have low physical activity and poor dietary quality [44,46].

In older adults, research is limited, but the findings suggest that dietary and health-related factors play a significant mediating role in the association between egg consumption and mortality. A 16-year follow-up of 521,120 US participants [21], aged in the range of 50–71 years, reported that, compared to the lowest level of whole-egg consumption, all additional levels of egg intake demonstrated associations with a significantly increased risk of all-cause mortality, CVD mortality, and cancer mortality, though these associations were not maintained when models were adjusted for dietary cholesterol, suggesting that dietary cholesterol may mediate the association between egg consumption and mortality. A prospective analysis in adults also observed similar trends [37,38,47]. These results align with Papanikolaou et al.’s [48] analysis of NHANES data, which found that egg consumers had higher nutrient intakes and better nutritional status; this was particularly pronounced with the addition of eggs in already high-quality diets, which reinforces the need for epidemiological research to control for dietary factors [13,14]. Our primary results for CVD mortality are maintained for those with moderate to high dietary quality, with a slightly lowered risk observed for those with a higher quality diet, suggesting that dietary quality may play a further protective role in the association between egg consumption and mortality, though it does not have the mediating influence that we observed from other cohorts. Importantly, this difference may be due to the lowered statistical power in the subgroup analysis.

A high heterogeneity across studies was reported by Mousavi et al. [19], with more pronounced mortality risks in studies adjusting for blood cholesterol, dyslipidemia, or dyslipidemia therapy, and those without longitudinal egg consumption measurements. This contrasts with our finding that the association between weekly egg consumption and lowered risk of CVD mortality held regardless of the presence of dyslipidemia. This may speak to changes in the health risks associated with higher cholesterol in those over the age of 70 y [49], but also aligns with current research that suggests no association between increased CVD risk profiles and dietary cholesterol associated with egg consumption [50,51]. In contrast, the nutritional profile of eggs may support CVD health. Eggs are a key source of omega-3 fatty acids [44], having been observed to lower triglycerides and lower the risk of coronary heart disease [52]. They also contain the key antioxidant proteins phosvitin, ovotransferrin, and ovalbumin that inhibit lipid oxidation [53]. Additionally, eggs are an important source of dietary choline, which has been demonstrated to inhibit atherosclerotic pathways [54] and has been associated with a lower risk of CVD [55].

### 4.1. Possible Implications for Dietary Guidelines

The Australian Dietary Guidelines [56] recommend adults consume up to 7 eggs per week, a recommendation mirrored by the American Heart Association (AHA) [16] for normocholesterolemic adults, though cautioned for those with dyslipidemia. Similar guidelines are observed in China [57] and Ireland [58]. However, many European countries suggest limiting egg consumption to 3–4 per week [58]. The AHA [16] supports up to 2 eggs per day for older adults with normal cholesterol levels, citing nutrient density and convenience. Our findings indicate that consuming eggs up to 6 times a week may be associated with a reduced risk of all-cause and CVD mortality in older adults and suggest potential age-related differences in mortality risk that may be important to inform dietary guidelines. Significant heterogeneity is reported within the wider evidence base, with differences in study methodology, dietary assessment, and adjustment for dietary factors all noted as limitations. These factors increase the risk of residual confounding and obstruct the comparison and reporting of clear conclusions on the moderating role of dietary cholesterol [13,59] and dietary quality [19,21,41] on the association between egg intake and mortality.

Future research that addresses methodological inconsistencies in the measurement of dietary quality, and controls for potential moderating factors, is needed to better understand this relationship.

### 4.2. Study Strengths and Limitations

The strengths of this study include its large sample size and minimal loss to follow-up. Death ascertainment was confirmed by two sources, minimizing detection bias. The subgroup analysis highlighted the moderating effect of overall diet quality, lowering the risk of confounding by dietary factors on the overall association between egg consumption and mortality.

However, there are limitations. Egg consumption was self-reported, risking recall bias. The type of egg, method preparation, and serving sizes were not captured, affecting the ability to attribute nutritional value of egg consumption within the overall diet [11], and serving sizes were also not recorded. Future studies would benefit from gathering more granular data on egg consumption. The number consuming eggs daily in this cohort was small, which may increase the risk of type 2 error. The analysis of the differences between the included and missing data was small, but significant differences in age, waist circumference, diabetes, and diet score between those with complete data and those with missing data suggest that those included may have a lower risk profile. Derived from a primary prevention trial, the study population consisted of independently living older Australians without independence-limiting chronic diseases or cardiovascular disease at enrolment in the ASPREE clinical trial. As a result, this group is a potentially healthier sample than the general similarly aged population from which they were drawn, which may limit the generalizability of this study. Studies in more representative populations of older cohorts may be beneficial to confirm the association between egg consumption and mortality in older adults. This is an observational study; so, despite adjusting for various confounders, some effects may have been underestimated or undetected, increasing the risk of residual confounding.

## 5. Conclusions

Our study found that, for relatively healthy older adults, the consumption of eggs 1–6 times per week was associated with a lower risk of all-cause mortality and CVD mortality compared to those who rarely or never eat eggs. No such potential benefit was observed with daily egg consumption. Our findings suggest that the association between egg consumption and CVD mortality is not mediated by dyslipidemia or dietary quality in older adults. These findings may be beneficial in the development of evidence-based dietary guidelines for older adults, though further longitudinal research is needed to confirm our findings and to better understand the mechanisms underlying these associations.

## Figures and Tables

**Table 1:** Baseline characteristics of 8756 community-dwelling adults aged 70 years and over, presented by the egg consumption frequency.

Covariates n (%)		Total (N = 8756)	Never/Infrequent Egg Consumption (n = 2119) 24.2%	Weekly Egg Consumption (n = 6414) 73.2%	Daily Egg Consumption (n = 223) 2.6%
Sex	Female	4725 (54.0)	1102 (52.0)	3515 (54.8)	108 (48.4)
Age Median (IQR)		76.9 (5.5)	77.21 (5.7)	76.8 (5.5)	77.01 (5.5)
IRSAD ^a^	1: Most Disadvantaged	1335 (15.2)	335 (16.0)	966 (15.0)	34 (15.2)
	2	1457 (16.6)	361 (17.0)	1054 (16.4)	42 (18.8)
	3	1605 (18.2)	382 (18.0)	1180 (18.4)	43 (19.3)
	4	1742 (20.0)	418 (19.6)	1280 (20.0)	44 (19.7)
	5: Least Disadvantaged	2975 (30.0)	623 (29.4)	1934 (30.2)	60 (27.0)
Education	≤12 years	5057 (57.7)	1282 (60.5)	3659 (57.1)	116 (52.0)
	>12 years	3699 (42.3)	837 (39.5)	2755 (42.9)	107 (48.0)
Physical Activity	Rarely/Never	137 (1.5)	46 (2.2)	89 (1.4)	2 (1.0)
	Low to Moderate	7432 (84.9)	1824 (86.1)	5429 (84.6)	179 (80.2)
Vigorous		1187 (13.6)	249 (11.7)	896 (14.0)	42 (18.8)
Smoking	Never	5770 (56.8)	1180 (55.7)	3669 (57.2)	123 (55.2)
	Former	4197 (40.9)	876 (41.3)	2613 (40.7)	94 (42.1)
	Current	236 (2.3)	63 (3.0)	132 (2.1)	6 (2.7)
Alcohol	Non-Drinker	2260 (25.2)	596 (28.1)	1561 (24.4)	49 (22.0)
	Within Guidelines	3438 (39.3)	766 (36.1)	2587 (40.3)	85 (38.1)
	Exceeds Guidelines	3112 (35.5)	757 (35.8)	2266 (35.3)	89 (39.9)
Waist Circumference, cm [Mean (SD)]		96 (12.6)	95.9 (12.7)	96.0 (12.7)	97.9 (11.2)
Hypertension	Yes	7588 (86.7)	1846 (87.1)	5555 (86.6)	187 (83.9)
Diabetes	Yes	1005 (11.5)	240 (11.3)	737 (11.5)	28 (12.6)
Dyslipidemia	Yes	7060 (80.6)	1691 (79.8)	5198 (81.0)	171 (76.7)
Polypharmacy	Yes	2014 (23.0)	505 (23.8)	1458 (22.7)	51 (22.9)
Frailty	Non-frail	4573 (52.2)	1069 (50.4)	3392 (52.9)	112 (50.2)
Score	Pre-Frail	3280 (37.5)	805 (38.0)	3292 (37.3)	83 (37.2)
	Frail	903 (10.3)	245 (11.6)	630 (9.8)	28 (12.6)
Self-reported	Poor	83 (1.0)	21 (1.0)	62 (1.0)	0 (0)
Oral Health	Fair/good	3768 (43.0)	934 (44.1)	2730 (42.5)	104 (46.6)
	V. good/excellent	4905 (56.0)	1164 (54.9)	4156 (56.5)	119 (53.4)
Depression ^b^	None	3705 (42.3)	899 (42.4)	2708 (42.2)	98 (43.9)
	Mild	3677 (42.0)	876 (41.3)	2710 (24.3)	91 (40.8)
	Moderate	1374 (15.7)	344 (16.3)	996 (15.5)	34 (15.3)
Diet	T1—Low	319 (3.6)	119 (5.6)	197 (3.1)	3 (1.3)
Score	T2—Moderate	5989 (68.4)	1572 (74.2)	4277 (66.7)	140 (62.8)
	T3—High	2448 (28.0)	428 (20.2)	1940 (30.2)	80 (35.9)
Treatment Arm					
	Placebo	4355 (49.7)	1040 (49.1)	3192 (49.8)	123 (55.2)
	Aspirin	4401 (50.3)	1079 (50.9)	3222 (50.2)	100 (44.8)

Values presented as n (%), except for age and waist circumference (mean, SD). ^a^ Depression symptoms assessed using the CESD. ^b^ Index of Relative Socio-economic Advantage and Disadvantage, Socio-economic Index for Areas, Australian Bureau of Statistics 2012.

**Table 2:** The association between egg consumption and all-cause and cause-specific mortality community-dwelling older adults: result of the Cox regression.

Egg Consumption	All-Cause Mortality HR [95% CI]	Cancer Mortality HR [95% CI]	CVD Mortality HR [95% CI]
Crude			
Never/infrequently	Ref	Ref	Ref
Weekly	0.77 [0.67–0.89]	0.89 [0.74–1.08]	0.65 [0.50–0.83]
Daily	1.20 [0.85–1.70]	1.21 [0.75–1.95]	1.29 [0.70–2.23]
Min. Adjusted ^a^			
Never/infrequently	Ref	Ref	Ref
Weekly	0.82 [0.71–0.93]	0.94 [0.78–1.13]	0.67 [0.53–0.87]
Daily	1.12 [0.79–1.58]	1.19 [0.74–1.91]	1.21 [0.66–2.22]
Fully Adjusted ^b^			
Never/infrequently	Ref	Ref	Ref
Weekly	**0.85 [0.74–0.98]**	0.93 [0.76–1.16]	**0.71 [0.54–0.91]**
Daily	1.20 [0.85–1.71]	1.36 [0.82–02.27]	1.43 [0.79–2.58]

HR: Hazard ratio, 95% CI: 95% Confidence intervals; **Bold**: indicates significant (<0.05) result; ^a^ Minimally adjusted model: Adjusted for age & Sex; ^b^ Fully adjusted model: Adjusted for sex, age, IRSAD, education, physical activity, smoking status, alcohol consumption, waist circumference, dyslipidemia, hypertension, type 2 diabetes, polypharmacy, depression (CES D-10), frailty score, self-reported oral health, and treatment arm.

**Table 3:** The association between egg consumption and all-cause and cause-specific mortality in community-dwelling older adults: fully adjusted results of Cox regression stratified by diet quality score and dyslipidemia.

Egg Consumption	All-Cause Mortality HR [95% CI]	Cancer Mortality HR [95% CI]	CVD Mortality HR [95% CI]
**Diet Quality Score Tertile ^a^**			
T1			
Infrequent/Never	Ref	Ref	Ref
Weekly	1.04 [0.58–1.86]	0.59 [0.23–1.44]	2.26 [0.73–7.00]
Daily	1.60 [0.18–13.85]	4.69 [0.32–68.05]	----
T2			
Infrequent/Never	Ref	Ref	Ref
Weekly	0.86 [0.73–1.01]	1.00 [0.78–1.28]	**0.67 [0.50–0.90]**
Daily	0.94 [0.60–1.51]	0.97 [0.47–2.02]	1.11 [0.51–2.44]
T3			
Infrequent/Never	Ref	Ref	Ref
Weekly	0.79 [0.57–1.08]	0.82 [0.50–1.34]	**0.56 [0.32–0.99]**
Daily	1.65 [0.92–2.94]	2.19 [0.94–5.06]	1.69 [0.65–4.43]
**Dyslipidemia ^b^**			
No			
Infrequent/Never	Ref	Ref	Ref
Weekly	0.79 [0.60–1.04]	0.85 [0.57–1.30]	**0.57 [0.33–0.97]**
Daily	1.14 [0.59–2.19]	1.02 [0.35–2.96]	2.43 [0.93–6.40]
Yes			
Infrequent/Never	Ref	Ref	Ref
Weekly	0.86 [0.74–1.01]	0.96 [0.75–1.2448]	**0.73 [0.54–0.97]**
Daily	1.15 [0.76–1.80]	1.48 [0.82–2.66]	1.00 [0.46–2.20]

HR: Hazard ratio, 95% CI: 95% Confidence intervals; **Bold**: indicates significant (<0.05) result ^a^ Adjusted for age and sex, IRSAD, education, physical activity, smoking status, alcohol consumption, waist circumference, polypharmacy, dyslipidemia, hypertension, type 2 diabetes, depression (CES-D-10), frailty score, self-reported oral health, and treatment arm. ^b^ Adjusted for: age, sex, IRSAD, education, physical activity, smoking status, alcohol consumption, waist circumference, polypharmacy, hypertension, type 2 diabetes, depression (CES-D-10), frailty score, self-reported oral health, diet quality score tertile, and treatment arm.

## Data Availability

Access to ASPREE and ALSOP data is available via application. Details can be found at: https://ams.aspree.org/public/ (accessed on 11 December 2024).

## Supplementary Materials

The following supporting information can be downloaded at: https://www.mdpi.com/article/10.3390/nu17020323/s1, Table S1: Mortality events stratified by the level of egg consumption; Table S2: The association between egg consumption and all-cause and cause-specific mortality in community-dwelling older adult: results of Cox regression via dietary quality score tertile; Table S3: The association between egg consumption and all-cause and cause-specific mortality in community-dwelling older adult: results of Cox regression via dyslipidemia; Table S4: Sensitivity analyses: Fine–Grey analysis association between egg consumption and mortality; Table S5: Sensitivity analyses: Fine–Grey analysis association between egg consumption and mortality stratified by diet quality score; Table S6: Sensitivity analyses: Fine–Grey analysis association between egg consumption and mortality stratified by dyslipidaemia.
