# Supplementary material for: Egg Consumption and Mortality: A Prospective Cohort Study of Australian Community-Dwelling Older Adults
Source: Nutrients. 2025 Jan 17;17(2):323. doi: 10.3390/nu17020323 (PMC11767731; doi:10.3390/nu17020323)
Supplement: Supplementary file 1 [file nutrients-17-00323-s001.zip › nutrients-3395001-supplementary.pdf]

Supplementary Material

Table S1. Mortality events stratified by level of egg consumption

**Mortality Events**		**Events (%) (N=8756)**	**Never/Infrequently egg consumption (n=2119)**	**Weekly egg consumption (n=6414)**	**Daily egg consumption (n=223)**
All-Cause Mortality	Total	1034 [11.8%]	295 [13.4%]	703 [11.0%]	36 [16.1%]
Cancer Mortality	Total	453 [5.2%]	120 [5.6%]	316 [4.9%]	17 [7.6%]
CVD Mortality	Total	292 [3.3%]	91 [4.3%]	174 [2.7%]	11 [4.9%]

Table S2. The association between egg consumption and all-cause and cause-specific mortality in Community - dwelling older adult: results of Cox regressionstratefied by diet quality score tertile

**Egg Consumption**	**All-Cause Mortality HR [95%CI]**	**Cancer Mortality HR [95%CI]**	**CVD Mortality HR [95%CI]**
Crude Model			
**Q1**<br>Infrequent<br>/Never<br>Weekly<br>Daily<br>**Q2**<br>Infrequent<br>/Never<br>Weekly<br>Daily<br>**Q3**<br>Infrequent<br>/Never<br>Weekly<br>Daily	 Ref<br>1.21 [0.72-2.03]<br>2.58 [0.34-19.23]<br><br>Ref<br>0.79 [0.67-0.92]<br>0.99 [0.62-1.58]<br><br>Ref<br>0.76 [0.56-.1.04]<br>1.80 [1.02-3.15]	 Ref<br>0.72 [0.32-1.62]<br>4.42 [0.56-34.35]<br><br>Ref<br>0.93 [0.73-1.19]<br>1.04 [0.50-2.15]<br><br>Ref<br>0.80 [0.49-1.29]<br>2.13 [0.94-4.82]	 Ref<br>2.07 [0.76-5.62]<br>--<br><br>Ref<br>0.63 [0.47-0.85]<br>1.00 [0.43-2.31]<br><br>Ref<br>0.57 [0.33-0.99]<br>1.97 [0.77-5.00]
Minimally Adjusted Model [a]			
**Q1**<br>Infrequent<br>/Never<br>Weekly<br>Daily<br>**Q2**<br>Infrequent<br>/Never<br>Weekly	 Ref<br>1.08 [0.64-1.82]<br>2.06 [0.27-15.70]<br><br>Ref<br>0.83 [0.70-0.97]<br>0.94 [0.59-1.50]	 Ref<br>0.59 [0.26-1.33]<br>2.41 [0.29-19.85]<br><br>Ref<br>0.97 [0.76-1.24]<br>1.00 [0.48-2.05]	 Ref<br>1.92 [0.70-5.34]<br>---<br><br>Ref<br>0.66 [0.49-0.89]<br>0.97 [0.42-2.23]

Daily<br>**Q3**<br>Infrequent<br>/Never<br>Weekly<br>Daily	Ref<br>0.78 [0.57-.1.08]<br>1.50 [0.85-2.63]	Ref<br>0.83 [0.51-1.34]<br>1.84 [0.81-4.18]	Ref<br>0.57 [0.32-1.00]<br>1.60 [0.63-4.09]
Fully Adjusted [b]			
**T1**<br>Infrequent<br>/Never<br>Weekly<br>Daily<br>**T2**<br>Infrequent<br>/Never<br>Weekly<br>Daily<br>**Q3**<br>Infrequent<br>/Never<br>Weekly<br>Daily	 Ref<br>1.04 [0.58-1.86]<br>1.60 [0.18-13.85]<br><br>Ref<br>0.86 [0.73-1.01]<br>0.94 [0.60-1.51]<br><br>Ref<br>0.79 [0.57-.1.08]<br>1.65 [0.92-2.94]	 Ref<br>0.59 [0.23-1.44]<br>4.69 [0.32-68.05]<br><br>Ref<br>1.00 [0.78-1.28]<br>0.97 [0.47-2.02]<br><br>Ref<br>0.82 [0.50-1.34]<br>2.19 [0.94-5.06]	 Ref<br>2.26 [0.73-7.00]<br>----<br><br>Ref<br>**0.67 [0.50-0.90]**<br>1.11 [0.51-2.44]<br><br>Ref<br>**0.56 [0.32-0.99]**<br>1.69 [0.65-4.43]

HR: Hazard Ratio, 95%CI : 95% Confidence intervals

a Minimally Adjusted Model: Adjusted for Age and Sex

b Fully Adjusted Model: Adjusted for IRSAD, education, physical activity, smoking status, alcohol consumption, waist circumference, dyslipidaemia, hypertension, type 2 diabetes, polypharmacy, depression (CES D-10), frailty score, self-reported oral health, Treatment Arm

Table S3. The association between egg consumption and all-cause and cause-specific mortality in Community-dwelling older adult: results of Cox regression stratrefied by dyslipidaemia

**Egg Consumption**	**All-Cause Mortality HR [95%CI]**	**Cancer Mortality HR [95%CI]**	**CVD Mortality HR [95%C]**
Crude Model			
**No**<br>Infrequent<br>/Never<br>Weekly<br>Daily<br><br>**Yes**<br>Infrequent<br>/Never<br>Weekly<br>Daily	 Ref<br>**0.74 [0.57-0.96]**<br>1.08 [0.57-2.03]<br><br><br>Ref<br>**0.79 [0.67-0.92]**<br>1.22 [0.80-1.85]	 Ref<br>0.74 [0.50-1.11]<br>0.92 [0.32-2.60]<br><br><br>Ref<br>0.91 [0.71-1.16]<br>1.58 [0.88-2.84]	 Ref<br>**0.55 [0.33-0.94]**<br>2.05 [0.83-5.05]<br><br><br>Ref<br>**0.68 [0.51-0.91]**<br>0.91 [0.39-2.11]
Minimally Adjusted Model [a]			
**No**<br>Infrequent<br>/Never<br>Weekly<br>Daily<br>**Yes**<br>Infrequent<br>/Never<br>Weekly<br>Daily	 Ref<br>**0.76 [0.58-0.99]**<br>1.03 [0.55-1.94]<br><br>Ref<br>**0.83 [0.70-0.97]**<br>1.13 [0.75-1.72]	 Ref<br>0.74 [0.50-1.14]<br>0.92 [0.31-2.45]<br><br>Ref<br>0.95 [0.74-1.21]<br>1.50 [0.84-2.69]	 Ref<br>**0.57 [0.34-0.96]**<br>2.05 [0.82-5.09]<br><br>Ref<br>**0.71 [0.53-0.95]**<br>0.83 [0.36-1.93]
Fully Adjusted [b]			
**No**<br>Infrequent<br>/Never<br>Weekly<br>Daily<br>**Yes**<br>Infrequent<br>/Never<br>Weekly<br>Daily	 Ref<br>0.79 [0.60-1.04]<br>1.14 [0.59-2.19]<br><br>Ref<br>0.86 [0.74-1.01]<br>1.15 [0.76-1.80]	 Ref<br>0.85 [0.57-1.30]<br>1.02 [0.35-2.96]<br><br>Ref<br>0.96 [0.75-1.2448]<br>1.48 [0.82-2.66]	 Ref<br>**0.57 [0.33-0.97]**<br>2.43 [0.93-6.40]<br><br>Ref<br>**0.73 [0.54-0.97]**<br>1.00 [0.46-2.20]

HR: Hazard Ratio, 95%CI : 95% Confidence intervals

a **Minimally Adjusted Model:** Adjusted for Age and Sex

b **Fully Adjusted Model:** Adjusted for IRSAD, education, physical activity, smoking status, alcohol consumption, waist circumference, hypertension, type 2 diabetes, polypharmacy, depression (CES D-10), frailty score, self-reported oral health. & diet quality score tertile. Treatment Arm

Table S4. Sensitivity Analyses: Fine Grey Analysis Association Between Egg Consumption and Mortality

**Egg Consumption**	***Cancer Mortality SHR^ [95%CI]**	***CVD Mortality SHR^[95%CI]**
**Crude**		
Crude		
Never/infrequently	Ref	Ref
Weekly	0.86 [0.71-1.07]	0.66 [0.51-0.84]
Daily	1.34 [0.82-2.19]	1.23 [0.69-2.22]
**Min Adjusted** [a]		
Never/infrequently		
Weekly	Ref	Ref
Daily	0.91 [0.75-1.12]	0.69 [0.54-0.89]
	1.32 [0.2-2.14]	1.23 [0.68-2.21]
**Fully Adjusted** [b]		
Never/infrequently		
Weekly	Ref	Ref
Daily	0.94 [0.77-1.16]	**0.72 [0.57-0.93]**
	1.39 [0.85-02.25]	1.46 [0.83-2.57]

^ Sub-Distribution Hazard Ratio

a Minimally Adjusted Model: Adjusted for Age and Sex

b Fully Adjusted Model: Adjusted for IRSAD, education, physical activity, smoking status, alcohol consumption, waist circumference, polypharmacy dyslipidaemia, hypertension, type 2 diabetes, depression (CES-D-10), frailty score, self-reported oral health. Treatment Arm

Table S5. Sensitivity Analyses: Fine Grey Analysis Association Between Egg Consumption and Mortality Stratified by Diet Quality Score Tertile

Egg Consumption	Cancer Mortality SHR^ [95%CI]	CVD Mortality SHR^[95%CI]
Crude Model		
**Q1**		
Infrequent /Never	Ref	Ref
Weekly	0.71 [0.33-1.54]	2.05 [0.77-5.42]
Daily	3.60 [0.65-19.7]	--
**Q2**		
Infrequent /Never	Ref	Ref
Weekly	0.94 [0.74-1.19]	0.64 [0.48-0.85]
Daily	1.02[0.50-2.06]	0.96 [0.42-2.17]
**Q3**		
Infrequent /Never	Ref	Ref
Weekly	0.80 [0.50-1.29]	0.58 [0.33-1.01]
Daily	2.03 [0.93-4.44]	1.88 [0.76-4.64]
Minimally Adjusted Model [a]		
**Q1**		
Infrequent /Never	Ref	Ref
Weekly	0.61 [0.29-1.28]	1.92 [0.70-5.22]
Daily	1.64 [0.35-7.56]	---
**Q2**		
Infrequent /Never	Ref	Ref
Weekly	0.98 [0.77-1.25]	0.68 [0.51-0.89]
Daily	1.00 [0.49-2.03]	0.95 [0.42-2.16]
**Q3**		
Infrequent /Never	Ref	Ref
Weekly	0.85 [0.53-1.35]	0.60 [0.35-1.04]
Daily	1.92 [0.91-4.07]	1.86 [0.77-4.49]
Fully Adjusted [b]		
**Q1**		
Infrequent /Never	Ref	Ref
Weekly	0.65 [0.29-1.45]	1.90 [0.70-5.30]
Daily	3.05 [0.45-20.15]	----
**Q2**		
Infrequent /Never	Ref	Ref
Weekly	1.01 [0.80-1.28]	**0.67 [0.51-0.90]**
Daily	1.01 [0.50-2.05]	1.14 [0.52-2.46]
**Q3**		
Infrequent /Never	Ref	Ref
Weekly	0.85 [0.53-1.35]	0.62 [0.35-1.10]
Daily	2.26 [1.07-4.75]	1.90 [0.77-4.69]

^ Sub-Distribution Hazard Ratio

a Minimally Adjusted Model: Adjusted for Age and Sex

b Fully Adjusted Model: Adjusted for IRSAD, education, polypharmacy physical activity, smoking status, alcohol consumption, waist circumference, dyslipidaemia, hypertension, type 2 diabetes, depression (CES-D-10), frailty score, self-reported oral health & treatment arm .

Table S6. Sensitivity Analyses: Fine Grey Analysis Association Between Egg . Consumption and Mortality Stratified by Dyslipidaemia

Dyslipidaemia	Cancer Mortality SHR [95%CI]	CVD Mortality SHR [95%CI]
Crude Model		
**No**		
Infrequent	Ref	Ref
/Never	0.76 [0.52-1.12]	**0.58 [0.35-0.96]**
Weekly	0.94 [0.34-2.54]	2.14 [0.91-5.03]
Daily		
**Yes**	Ref	Ref
Infrequent	0.91 [0.72-1.16]	**0.68 [0.51-0.91]**
/Never	1.51 [0.86-2.65]	0.85 [0.37-1.95]
Weekly		
Daily		
Minimally Adjusted Model [a]		
**No**		
Infrequent	Ref	Ref
/Never	0.80 [0.65-1.16]	0.61 [0.36-1.00]
Weekly	0.96 [0.36-2.57]	**2.36 [1.01-5.50]**
Daily		
**Yes**	Ref	Ref
Infrequent	0.96 [0.75-1.22]	**0.73 [0.55-0.96]**
/Never	1.47 [0.84-2.57]	0.82 [0.36-1.87]
Weekly		
Daily		
Fully Adjusted [b]		
**No**		
Infrequent	Ref	Ref
/Never	0.89 [0.60-1.30],	0.61 [0.37-1.02]
Weekly	1.17 [0.45-3.06],	**3.01 [1.25-7.23]**
Daily		
**Yes**	Ref	Ref
Infrequent	0.97 [0.77-1.24]	**0.73 [0.56-0.97]**
/Never	1.47 [0.84-2.59],	1.00 [0.47-2.12]
Weekly		
Daily		

^ Sub-Distribution Hazard Ratio

a Minimally Adjusted Model: Adjusted for Age & Sex

b Fully Adjusted Model: Adjusted for IRSAD, education, physical activity, smoking status, alcohol consumption, waist circumference, polypharmacy hypertension, type 2 diabetes, depression (CES-D-10), frailty score, self-reported oral healthdiet quality score tertile & treatment arm.
